# Nitazoxanide Inhibits the Bifunctional Enzyme GlG6PD::6PGL of *Giardia lamblia*: Biochemical and In Silico Characterization of a New Druggable Target

**DOI:** 10.3390/ijms241411516

**Published:** 2023-07-15

**Authors:** Víctor Martínez-Rosas, Beatriz Hernández-Ochoa, Laura Morales-Luna, Daniel Ortega-Cuellar, Abigail González-Valdez, Roberto Arreguin-Espinosa, Yadira Rufino-González, Ernesto Calderón-Jaimes, Rosa Angélica Castillo-Rodríguez, Carlos Wong-Baeza, Isabel Baeza-Ramírez, Verónica Pérez de la Cruz, Abraham Vidal-Limón, Saúl Gómez-Manzo

**Affiliations:** 1 Laboratorio de Bioquímica Genética, Instituto Nacional de Pediatría, Secretaría de Salud, Mexico City 04530, Mexico; ing_vicmr@hotmail.com (V.M.-R.); lauraeloisamorales@gmail.com (L.M.-L.); 2 Programa de Posgrado en Biomedicina y Biotecnología Molecular, Escuela Nacional de Ciencias Biológicas, Instituto Politécnico Nacional, Mexico City 11340, Mexico; 3 Laboratorio de Inmunoquímica, Hospital Infantil de México Federico Gómez, Secretaría de Salud, Mexico City 06720, Mexico; beatrizhb_16@ciencias.unam.mx (B.H.-O.); ecalderj5@yahoo.com.mx (E.C.-J.); 4 Posgrado en Ciencias Biológicas, Universidad Nacional Autónoma de México, Mexico City 04510, Mexico; 5 Laboratorio de Nutrición Experimental, Instituto Nacional de Pediatría, Secretaría de Salud, Mexico City 04530, Mexico; dortegadan@gmail.com; 6 Departamento de Biología Molecular y Biotecnología, Instituto de Investigaciones Biomédicas, Universidad Nacional Autónoma de México, Mexico City 04510, Mexico; abigaila@biomedicas.unam.mx; 7 Departamento de Química de Biomacromoléculas, Instituto de Química, Universidad Nacional Autónoma de México, Mexico City 04510, Mexico; arrespin@unam.mx; 8 Laboratorio de Parasitología Experimental, Instituto Nacional de Pediatría, Secretaría de Salud, Mexico City 04530, Mexico; yadirg@gmail.com; 9 CICATA Unidad Morelos, Instituto Politécnico Nacional, Boulevard de la Tecnología, 1036 Z-1, P 2/2, Atlacholoaya 62790, Mexico; racastillo@ipn.mx; 10 Laboratorio de Biomembranas, Departamento de Bioquímica, Escuela Nacional de Ciencias Biológicas, Instituto Politécnico Nacional, Mexico City 11340, Mexico; charlywong@icloud.com (C.W.-B.); isabelbaeza@yahoo.com (I.B.-R.); 11 Neurobiochemistry and Behavior Laboratory, National Institute of Neurology and Neurosurgery “Manuel Velasco Suárez”, Mexico City 14269, Mexico; veped@yahoo.com.mx; 12 Red de Estudios Moleculares Avanzados, Clúster Científico y Tecnológico BioMimic®, Instituto de Ecología A.C. (INECOL), Carretera Antigua a Coatepec 351, El Haya, Xalapa 91073, Mexico

**Keywords:** molecular dynamics simulations, polypharmacology, inhibition, G6PD::6PGL

## Abstract

Giardiasis, which is caused by *Giardia lamblia* infection, is a relevant cause of morbidity and mortality worldwide. Because no vaccines are currently available to treat giardiasis, chemotherapeutic drugs are the main options for controlling infection. Evidence has shown that the nitro drug nitazoxanide (NTZ) is a commonly prescribed treatment for giardiasis; however, the mechanisms underlying NTZ’s antigiardial activity are not well-understood. Herein, we identified the glucose-6-phosphate::6-phosphogluconate dehydrogenase (GlG6PD::6PGL) fused enzyme as a nitazoxanide target, as NTZ behaves as a GlG6PD::6PGL catalytic inhibitor. Furthermore, fluorescence assays suggest alterations in the stability of GlG6PD::6PGL protein, whereas the results indicate a loss of catalytic activity due to conformational and folding changes. Molecular docking and dynamic simulation studies suggest a model of NTZ binding on the active site of the G6PD domain and near the structural NADP^+^ binding site. The findings of this study provide a novel mechanistic basis and strategy for the antigiardial activity of the NTZ drug.

## 1. Introduction

Nitazoxanide (NTZ) (2-acetyloloxy-*N*-(5-nitro-2thiazolyl)benzamide) belongs to the family of drugs known as thiazolides, which consist of a nitrothiazole part and a salicylic acid moiety, connected via a peptide bond [1]. In vivo, the NTZ is rapidly deacetylated to tizoxanide (TIZ), a compound with equal effectiveness [2]. Initially, NTZ was developed as an antiparasitic drug, especially against *Cryptosporidium* spp. and *Giardia lamblia* (*G. lamblia*) [3], as well as other parasites [1,4,5]. Moreover, NTZ has been used in anaerobic bacteria such as *Helicobacter pylori*, *Bacteroides* species, and *Clostridium* species [6,7]. Antiviral activities have also been reported for this drug, including activity against influenza A and SARS-CoV-2 [8,9].

In vitro analyses of *G. lamblia* trophozoites showed that NTZ exerts essentially differential morphological and ultrastructural alterations on this parasite [10]; in the same study, some nitazoxanide derivatives were examined for antigiardial activity, demonstrating that the thiazole-associated nitro group is likely crucial for the in vitro activity of NTZ [10].

Several explanations have been proposed for the antiprotozoal effect of NTZ. For example, one such explanation suggests that NTZ provokes the noncompetitive inhibition of pyruvate: ferredoxin oxidoreductase (PFOR), an enzyme essential for anaerobic energy metabolism, which is present in amitochondriate human parasites and most anaerobic bacteria, but is not found in mammals. However, NTZ can directly kill bacteria (e.g., *Mycobacterium tuberculosis*) via a PFOR-independent mechanism, if PFOR is not known to be present in the given organism [11]. Another mechanism described for aerobic bacteria suggests that NTZ acts as an uncoupler, disrupting membrane potential and leading to an alteration of pH homeostasis [11]. Therefore, the mechanisms that underlie the antibacterial activity of NTZ are not well-understood. Several in vitro studies have proposed that the mechanism of action of MTZ (nitro drug) in *G. lamblia* depends on three enzymes—pyruvate ferredoxin oxidoreductase (PFOR), NADPH-dependent thioredoxin reductase (TrxR), and nitroreductase 1 (NR1)—which activate MTZ by reducing its nitro group to form a toxic, short-lived radical intermediate, causing DNA damage [12,13,14,15,16]. Regarding the mechanisms of action proposed for NTZ, it has been reported that recombinant NR-1 is capable of reducing NTZ compounds [17], and overexpression of the NR-1 enzyme in the WB *Giardia lamblia* strain increased NTZ susceptibility [18]. Furthermore, it has been observed that, in NTZ-resistant laboratory *Giardia lamblia* strains (WB-C4 and NTZII,) the gene encoding NR-1 is downregulated [19,20,21], suggesting that NR-1 reduces NTZ to toxic intermediates in *G. lamblia*, and that resistant lines avoid this effect by downregulating *nr-1* transcription. It has also been reported that NTZ may act as a steric inhibitor of protein disulfide isomerase (PDI) and PFOR enzymes [19,22]. In ABZ, the tubulin is implicated as the target of this drug in *G. lamblia*. The ABZ binds to β-tubulin structures, and cause deleterious effects on its microtubule-based cytoskeleton.

Our research group has explored the use of molecules with antiparasitic potential to disrupt the energetic metabolism of some pathogenic microorganisms (*G. lamblia* and *Trichomonas vaginalis*), including drugs that inhibit the glucose-6-phosphate::6-phosphogluconate dehydrogenase fused enzyme, a bifunctional enzyme involved in the pentose phosphate pathway (PPP), consequently inhibiting the proliferation of the mentioned microorganisms. Specifically, we reported that two compound analogs of nitazoxanide (CNZ-7 and CNZ-8) can inhibit the GlG6PD::6PGL from *G. lamblia* [23]. Although nitazoxanide analogs are effective inhibitors of GlG6PD::6PGL, such compounds are not commercially available drugs. In the present work, we determine whether nitazoxanide, an FDA-approved oral antiprotozoal drug for the treatment of diarrhea caused by *G. lamblia* with good bioavailability and safety, affects the activity of GlG6PD::6PGL. The results of inhibition assays show that NTZ inhibits the activity of GlG6PD::6PGL form *G. lamblia*. Additionally, molecular docking and dynamic simulation studies suggest that NTZ binds with high affinity to GlG6PD::6PGL and alters its stability. These results led us to propose a new pharmacological target for the NTZ broad-spectrum drug.

## 2. Results and Discussion

### 2.1. In Vitro Analysis of Fused GlG6PD::6PGL Inactivation with Antigiardial Drugs

**Figure 1:**
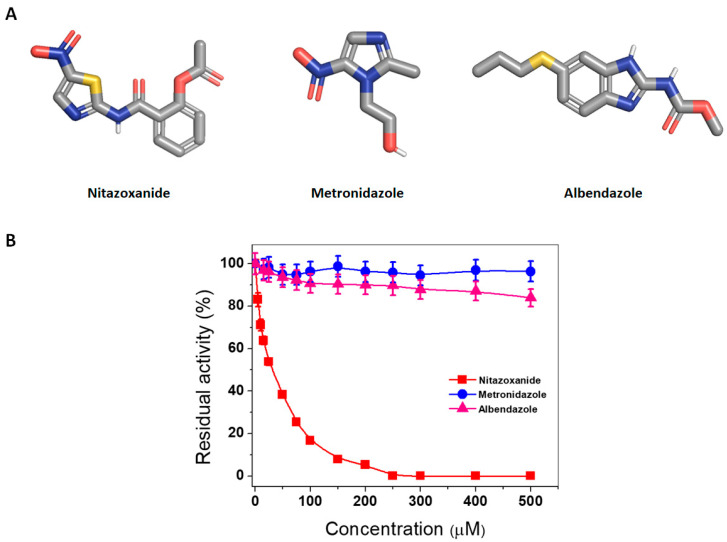
GlG6PD::6PGL enzyme inactivation assays. (**A**) Nitazoxanide (2-acetyloloxy-*N*-(5-nitro-2thiazolyl)benzamide) consist of a nitrothiazole ring and a salicylic acid moiety connected by a peptide bond. Metronidazole (2-(2-methyl-5-nitroimidazol-1-yl)ethanol) is a nitroimidazole. Albendazole (methyl *N*-(6-propylsulfanyl-1*H*-benzimidazol-2-yl)carbamate) is a benzimidazole. (**B**) Effects of antigiardial drugs nitazoxanide (NTZ), metronidazole (MTZ), and albendazole (ABZ) on the activity of the GlG6PD::6PGL enzyme. The assay was performed in triplicate, and the data represent the mean ± standard error.

Nitro heterocyclic compounds are used as therapeutic agents against a variety of protozoan and bacterial infections, but the exact mechanisms by which they induce microorganism death are unknown. For this reason, we evaluated whether three antigiardial drugs—two nitro drugs, NTZ and metronidazole (MTZ), and benzimidazole albendazole (ABZ)—alter the activity of fused GlG6PD::6PGL enzymes from *G. lamblia*. The structure of drugs is shown in Figure 1A, and the proposed mechanisms of action for these drugs are different. As shown in Figure 1B, only NTZ caused inhibition of the GlG6PD::6PGL enzyme in a concentration-dependent manner. As the concentration of NTZ increased, the activity of the GlG6PD::6PGL enzyme gradually decreased (Figure 1B). The IC_50_ value calculated for NTZ was 40 µM, whereas MTZ and ABZ did not have an effect at any tested concentration (Figure 1). These results suggest that the GlG6PD::6PGL enzyme is a target of NTZ, but not of MTZ or ABZ, at least at the tested concentrations.

**Figure 2:**
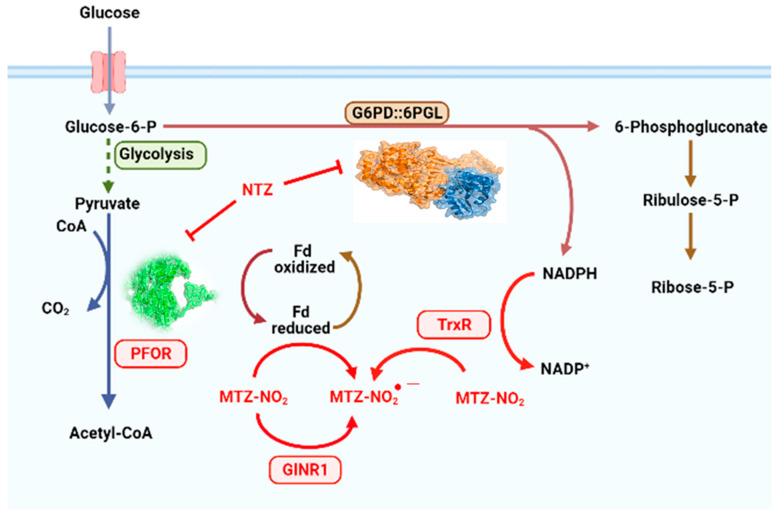
Mechanisms of action of metronidazole and nitazoxanide. The mechanism of action of metronidazole (MTZ-NO_2_) in *G. lamblia* depends on three enzymes—pyruvate ferredoxin oxidoreductase (PFOR), NADPH-dependent thioredoxin reductase (TrxR) and nitroreductase 1 (GlNR1)—which reduce MTZ to nitro radicals (MTZ-NO_2_^●−^), whereas nitazoxanide (NTZ) acts as an inhibitor of PFOR and G6PD::6PGL enzymes.

The inhibition of enzymatic activity of GlG6PD::6PGL fused enzyme by these drugs is interesting and differential. Although NTZ has been proposed to disrupt energy metabolism through the inhibition of PFOR and PDI enzymes [19,24,25] and, by its interaction with nitroreductases (GlNR1 and GlNR2) [16,21], our results indicate that the antigiardial drug NTZ also acts as an inhibitor on the fused G6PD::6PGL enzyme of *G. lamblia*. The fact that NTZ inhibits G6PD::GPGL suggests that the drug alters the redox metabolism of the parasite, representing a new mechanism by which NTZ has an antigiardiasis effect (Figure 2). In accordance with our findings, Carlos Nava-Zuazo et al. [26] reported that 2-acylamino-5-nitro-1,3-thiazole derivatives (nitazoxanide analogs) showed antigiardial activity. More recently, Morales-Luna et al. [23] found that two derivates of NTZ synthesized by Nava-Zuazo, named CNZ-7 and CNZ-8, reduced the activity of fused G6PD::6PGL enzyme of *G. lamblia*, with IC_50_ values of 150 and 80 µM, respectively.

### 2.2. Second-Order Rate Constant (k_2_) of NTZ

**Figure 3:**
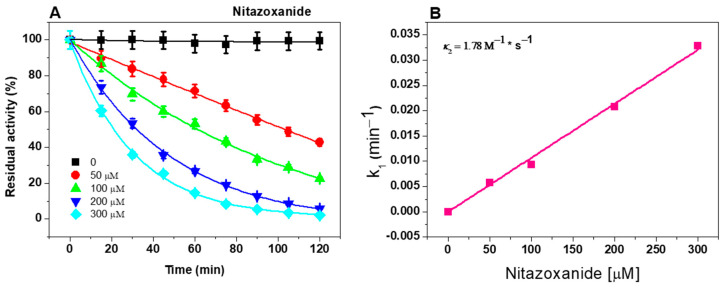
Inactivation of the fused GlG6PD::6PGL enzyme by NTZ. (**A**) The GlG6PD::6PGL enzyme was incubated with different concentrations of NTZ, and the residual activity was measured at the indicated times. (**B**) The second-order inactivation rate constant (*k*_2_) was obtained by fitting the calculated *k*_1_ value relative to the compound concentration and fit to a linear regression model. All experiments were carried out in triplicate; standard errors were less than 5%.

The next step was to determine the second-order inactivation rate constant (*k*_2_), which represents the reactivity rate of a compound to inactivate the enzyme, for NTZ based on the inactivation data. NTZ was the only antigiardial drug that inhibited the fused GlG6PD::6PGL. As shown in Figure 3A, the profile of the GlG6PD::6PGL enzyme inactivated by NTZ exhibited a negative effect on the catalytic activity. First, the initial velocities at four fixed compound concentrations at intervals between 0 and 120 min were determined, and the pseudo-first-order inactivation constants (*k*_1_) were calculated, representing the formation rate of the enzyme inactivator. Thereafter, the *k*_1_ values were plotted relative to the NTZ concentrations, and the second-order inactivation rate constant (*k*_2_) value of 1.78 M^−1^ * s^−1^ was calculated. The results suggest that NTZ presented a better *k*_2_ value regarding NTZ derivatives than CNZ-7 and CNZ-8, where *k*_2_ values of 0.97 M^−1^ * s^−1^ and 0.60 M^−1^ * s^−1^, respectively, were determined, indicating that NTZ presents a better formation ratio of the enzyme-inhibitor complex, and that the equilibrium shifts faster to the formation of an inactive enzyme. These results suggest that NTZ not only interacts with the PFOR, PDI, GlNR1, and GlNR2 proteins, but that it also has the ability to bind to the fused GlG6PD::6PGL enzyme of *G. lamblia*, which may contribute to the death of the parasite. Considering that NTZ is a broad-spectrum drug, it may act on multiple pharmacological targets to exert its therapeutic effect.

### 2.3. Intrinsic and Extrinsic Fluorescence Assays

**Figure 4:**
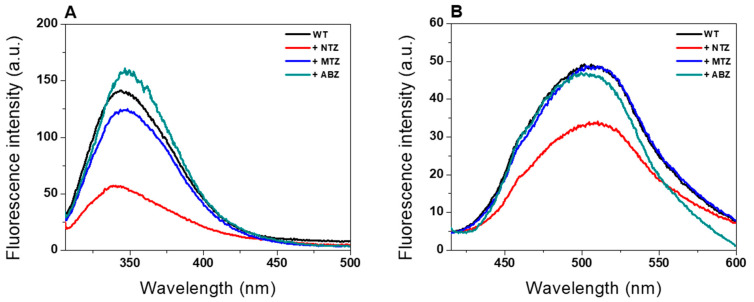
Fluorescence emission spectra of the GlG6PD::6PGL enzyme. (**A**) Intrinsic fluorescence and (**B**) extrinsic fluorescence ANS assays of the enzyme-free GlG6PD::6PGL or with NTZ, MTZ, and ABZ. The protein was adjusted to 0.1 mg/mL and incubated with 100 µM of each of the antigiardial drugs. Before measurement, the protein was incubated for 2 h at 37 °C. The data presented are the means of at least four independent experiments.

Changes in the tertiary structure and global stability of the fused GlG6PD::6PGL protein in the presence of NTZ, MTZ, and ABZ antigiardial drugs were evaluated using MTZ and ABZ as controls, because they did not inhibit the fused enzyme. We assessed the fluorescence properties of tryptophan residues present in the monomeric GlG6PD::6PGL for the intrinsic fluorescence assay. As shown in Figure 4A, the native protein showed a maximum fluorescence intensity at 339 nm, with a value of 140 arbitrary units (a.u.). However, in the presence of NTZ, a reduction of around 60% was observed with respect to the initial signal intensity in the maximum fluorescence (maximum fluorescence intensity of 57 a.u.). A blue shift in the fluorescence intensity occurs when the NTZ is present, with a maximal peak at 339 nm. Similar results were also observed with the compounds CNZ-7 and CNZ-8, which are derived from NTZ [23]. These results indicate that the protein aggregates (blue shift), and a rearrangement in the microenvironment of the tryptophan residues were observed after the incubation with NTZ, suggesting alterations in the global stability of the GlG6PD::6PGL protein. As expected, the MTZ (124 a.u.) and ABZ (158 a.u.) showed the same fluorescence intensity with respect to the GlG6PD::6PGL enzyme-free compound (140 a.u.), because they did not inhibit the fused enzyme.

Finally, an extrinsic fluorescence assay was used to corroborate the alterations in the tridimensional (3D) structure of the GlG6PD::6PGL protein in the presence of antigiardial drugs. We used the fluorescence of the ANS reagent, a molecule with a high affinity for hydrophobic regions, making it a sensitive indicator of protein folding and conformational changes. As shown in Figure 4B, the GlG6PD::6PGL protein-free drug and GlG6PD::6PGL in the presence of MTZ and ABZ showed the same pattern of extrinsic fluorescence, with a value of 50 a.u., whereas in the presence of NTZ, a decrease of around 50% in the extrinsic fluorescence intensity was observed relative to the GlG6PD::6PGL enzyme from *G. lamblia* free drugs. This decay in extrinsic fluorescence intensity indicates that NTZ induced conformational and folding changes in the protein, explaining the loss of catalytic activity.

### 2.4. Molecular Docking Study 

**Figure 5:**
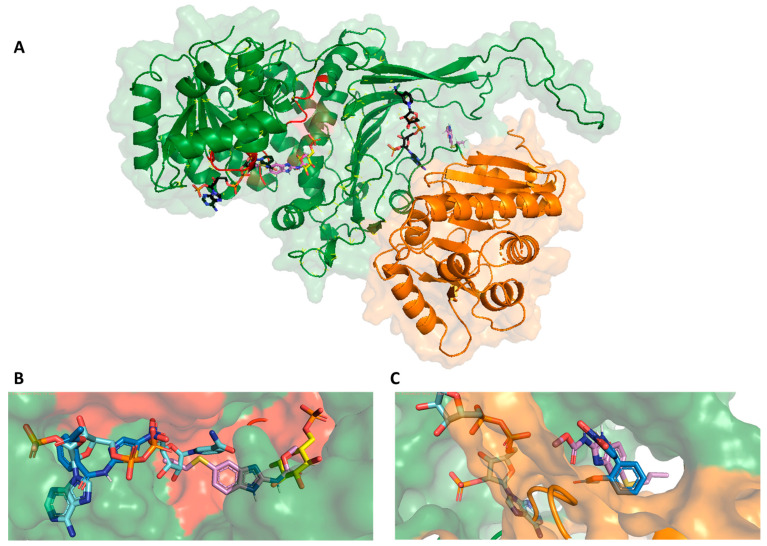
Prediction of the interaction zones between NTZ and ABZ on the GlG6PD::6PGL protein. (**A**) General view of the binding sites of NTZ and ABZ with GlG6PD::6PGL. (**B**) Closer view of the orthosteric site (near active site) of the G6PD domain. (**C**) Structural NADP^+^ binding site zone. The NADP^+^ and G6P molecules are shown in light blue and yellow color, respectively; NTZ and ABZ are shown in blue and violet, respectively.

A molecular docking study was conducted to explain the inhibitory activity shown by the NTZ drug on the GlG6PD::6PGL enzyme and to determine the possible binding mode in the fused enzyme. In addition, we selected ABZ to also carry out in silico studies, a drug with a different mechanism of action than NTZ, and which in the present work did not inhibit the G6PD::6PGL enzyme, thus allowing us to compare the results with the NTZ inhibitor. The results revealed a relationship between NTZ and ABZ for two main zones in the G6PD domain; the first zone was the near orthosteric site (active site) of the G6PD domain, and the second zone was near the structural NADP^+^ binding site (Figure 5A). NTZ molecules showed good affinity against both zones, with a free binding energy of –7.4 kcal/mol (*k*_2_ = 1.78 M^−1^ * s^−1^), whereas ABZ showed a better affinity for the active site of the enzyme, with a binding free energy of −7.6 kcal/mol. In contrast, the binding free energy in the structural NADP^+^ zone was −6.7 kcal/mol.

The fact that both drugs showed an affinity for the same zones may indicate that the affinity towards the two drugs is similar. Figure 5B shows the binding mode of NTZ and ABZ to the orthosteric site of the G6PD domain. As shown in the figure, NTZ is positioned close to the NADP^+^ molecule, and the binding mode analysis of NTZ shows that the nitro group of the thiazole ring directly interacts with phosphate group of NADP^+^. In contrast, ABZ (not an inhibitor of GlG6PD::6PGL) is positioned closer to the G6P substrate. The results published by other research groups have demonstrated that the nitro group of NTZ is important for the inhibitory activity on PFOR, as it interacts with and abstracts a proton from thiamine pyrophosphate (TPP), inactivating the catalytic cycle [22,27,28]. Substitution with bromine has also been demonstrated to result in a loss of activity against anaerobic bacteria [29]. Finally, Figure 5C shows the binding mode of NTZ and ABZ in the structural NADP^+^ binding site. The binding modes are similar; however, the NTZ showed better binding energy than ABZ.

**Figure 6:**
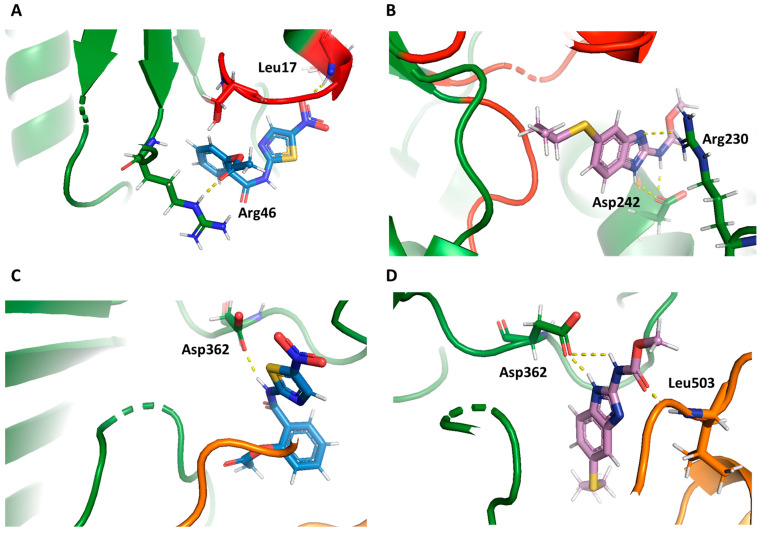
Interaction zones of NTZ and ABZ drugs on GlG6PD::6PGL protein. (**A**) Close up of the NADP^+^ catalytic site of GlG6PD::6PGL with NTZ and (**B**) ABZ. (**C**) Close-up of the NADP^+^ structural site of GlG6PD::6PGL with NTZ; and (**D**) ABZ. The NTZ and ABZ compounds are shown in blue and violet, respectively. H bonds are represented by dotted lines, amino acids are indicated by bold letters.

Docking calculations showed that the nitro thiazole moiety of NTZ entered into the NADP^+^ binding pocket of the G6PD domain, and that the nitro group formed two hydrogen bonds with Arg46 and Leu17, the latter of which is a crucial residue for correct NADP^+^ binding (Figure 6A), suggesting that the presence of NTZ affects the correct positioning of the NADP^+^ substrate, resulting in a decrease in activity. The ABZ compound exhibited none of these relevant interactions, which correlates with the low inhibitory activity reported in in vitro studies involving GlG6PD::6PGL (Figure 6B). Concerning the zone near the structural NADP^+^, NTZ and ABZ displayed a similar conformation (Figure 6C,D). However, the nitro thiazole moiety and nitro group of NTZ were properly oriented into a pocket, and the molecular docking binding energies calculated for NTZ (−7.4 kcal/mol) were better than those calculated for ABZ (−6.7 kcal/mol). These results are in agreement with those reported in experimental studies, which revealed that the antiprotozoal, antiproliferative, and anti-infective activities of nitazoxanide can be attributed to the nitro thiazole moiety [30,31], and that the salicylamide moiety exerts analgesic, antioxidant, and anti-infective effects, in addition to regulating immune responses [32].

### 2.5. Dynamic Behavior of GlG6PD::6PGL in Complex with Antigiardial Drugs

Molecular dynamics simulations (MDSs) can be a valuable tool for the investigation of large molecular motions and events with long durations, such as protein flexibility and large-scale conformational transitions [33,34]. Because both experimental and computational studies [35] have revealed that GlG6PD::6PGL is a rigid protein, we aimed to calculate the effect of drug binding on protein conformation using MDSs. Three independent MDSs were carried out to examine the binding modes between GlG6PD::6PGL protein in complex with NADP^+^, NTZ, ABZ (control), or a combination of NADP^+^ and drugs. Moreover, these data enable identification of localized regions with changes at the protein folding level.

To enhance the molecular sampling of substrate–enzyme conformations, 500 ns of Gaussian accelerated molecular dynamics simulations were applied to all systems to promote the appearance of molecular processes energetically distant from or dependent on conformational changes [36], using AMBER20 software and the ff14SB force field.

**Figure 7:**
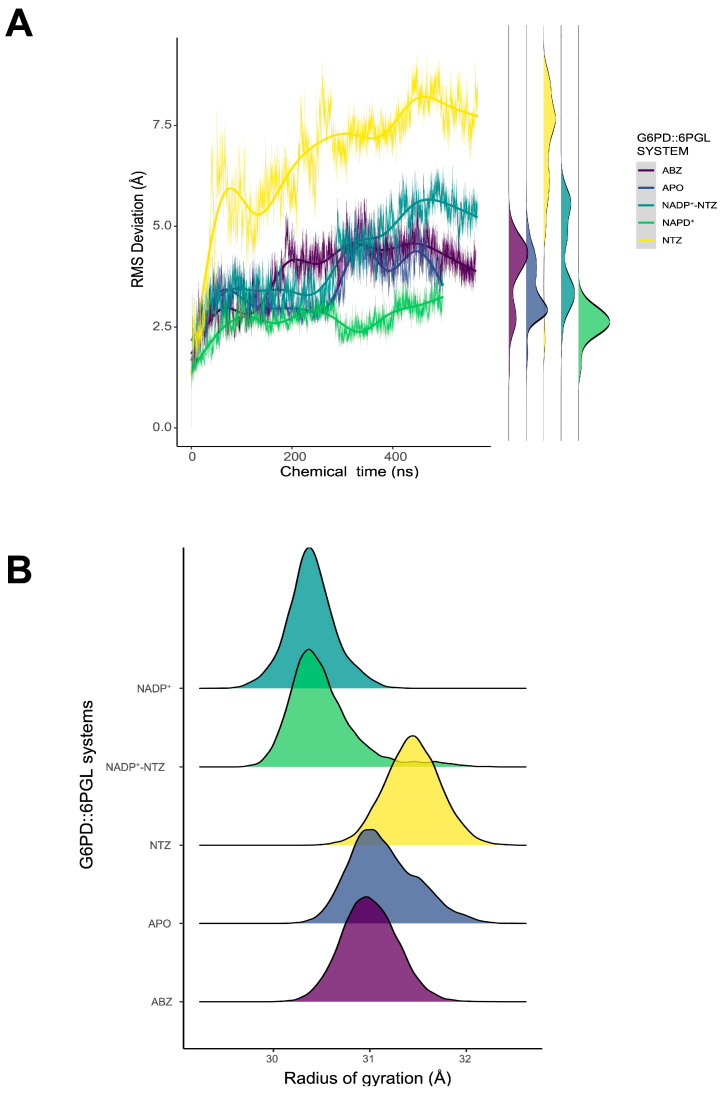
Dynamic behavior of GlG6PD::6PGL antiparasitic drug complexes. (**A**) Time series of alpha carbon (Cα) RMSD values for all complexes. The cumulative histogram of RMSD distribution for each complex is plotted on the side. (**B**) Comparison of the radius of gyration (Rog) of each complex.

Analysis of trajectories shows that the calculated binding position of both drugs promotes structural changes in the overall protein structure. The alpha carbon RMSD values for APO and ABZ complexes without NADP^+^ showed similar deviations to those of complexes with NADP+ in both sites. The presence of only NADP^+^ promoted a decrease in RMSD values as a structural stabilizing force. In contrast, NTZ complexes without NADP^+^, either in the cofactor or structural sites, promoted significant changes with increasing RMSD values ~7.5 Å (Figure 7A). In the NTZ complex with NADP^+^, the antiparasitic drug promoted an incipient destabilization, as shown by a slight increase in RMSD and the appearance of higher deviation distributions (Figure 7A, side panel).

**Figure 8:**
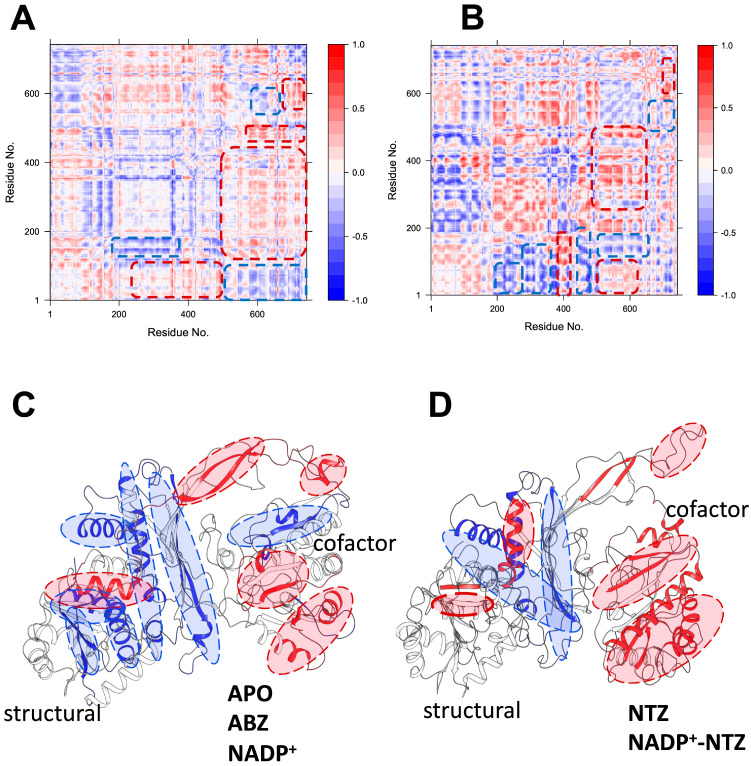
Correlated and anticorrelated GlG6PD::6PGL fluctuations and DCCM (cij) for complexes. (**A**,**B**) Representative color map of DCCM in the plane of all residue pairs evaluated for APO, ABZ NADP^+^, and NADP^+^-NTZ. The higher correlation of cij for residue pairs i–j is reflected by the colors in the DCCM closer to red, whereas colors close to blue indicate a low correlation (cij ∼ 0). (**C**,**D**) Average correlated GlG6PD::6PGL regions for APO, ABZ, and NADP^+^-NTZ, respectively.

Despite the changes in RMSD, the radius of gyration (Rog) remained around values ca. 31 Å for all systems, whereas for NTZ, the value slightly increased by ~32 Å. Because Rog is a metric that can be applied to evaluate the degree of folding persistence, the stabilizing effect of NADP^+^ on the overall folding is clear. Moreover, for NADP^+^–NTZ complexes, Rog values are similar to that of the NADP^+^-only complex (Figure 8B). However, the behavior changes when NADP^+^ is absent from the simulation and only NTZ is available for both cofactor and structural sites. These data suggest that NTZ can promote structural changes in localized protein regions with considerable flexibility.

A vector decomposition method was applied to measure the motion correlation between protein residue pairs to gain insight into the susceptible protein regions. Dynamic cross-correlation coefficient maps (DCCMs) were calculated for all complexes, representing the correlation of fluctuations (time-averaged vectors) between all regions, compared to a reference structure. The DCCM is expected to extract the dynamics of protein structures and help to identify changes in the motion (rigid or flexible domains) in G6PG::6PGL antiparasitic drug complexes.

**Figure 9:**
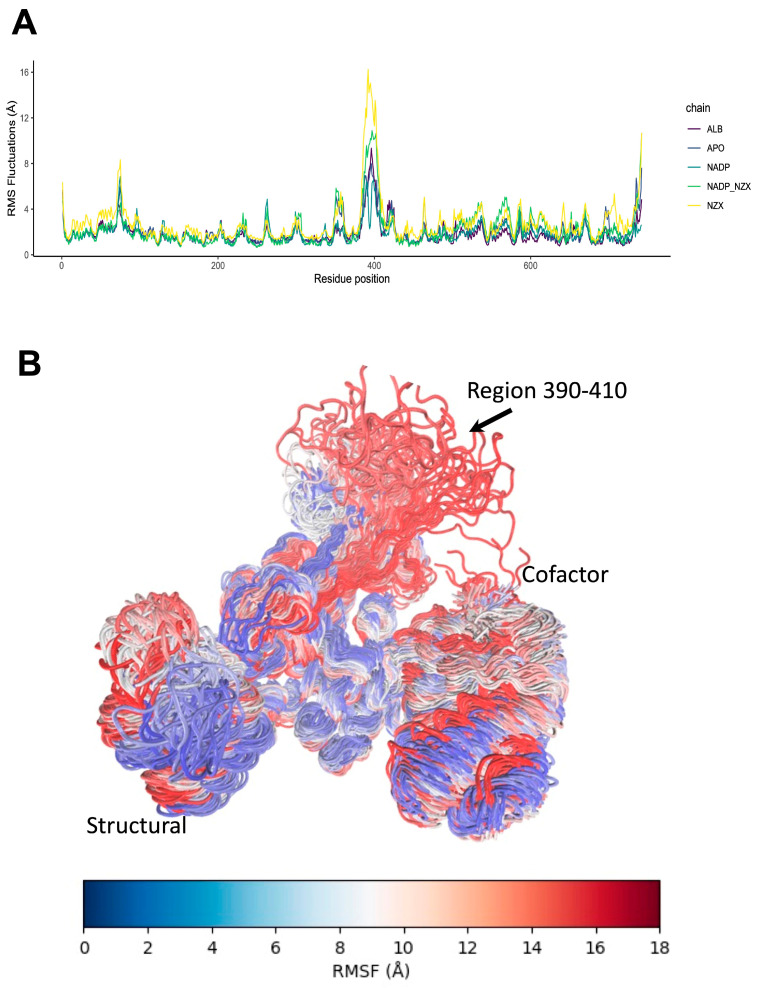
Dynamic fluctuations of G6PD::6PGL antigiardial drug complexes. (**A**) Time series of beta carbon (C_β_) RMSF by residue. (**B**) Ensemble of flexible region proteins. RMSF values are displayed as cofactors. Most flexible region (390-410) is indicated with an arrow.

Simulations of GlG6PD::6PGL complexes in APO, ABZ, and NADP^+^ indicate a positive coupling of the motions in cofactor site motions, which are depicted as red zones (Figure 8A,B). These motions are positively coupled with the fluctuations in a distal alpha-helical region 10 Å away, but in close contact with the structural site (Figure 8C). This arrangement enables the cofactor to fluctuate, depending on whether NADP^+^ is present in the structural site. Anticorrelated motions around structural sites are depicted as blue zones, and represent independent fluctuations not influenced by changes in the red zones. DCCM analysis shows that NTZ promotes subtle changes in the motions of protein folding. For example, the cofactor site motions are more correlated, suggesting that changes in the distal beta-sheet region can drastically impact the cofactor dynamics. Moreover, in the presence of NTZ, structural site-independent motions are reduced, indicating a decoupling of the central beta-sheet core from the rest of the protein (Figure 8D). All these changes in the motions of the GlG6PD::6PGL structures are also evidenced as changes in the RMSF of beta carbon atoms, where sidechain groups show modified patterns of fluctuations as a response to NTZ interactions (Figure 9A,B). Interestingly, the region just before residue 400 showed flexibility, which is allocated near the G6PD NADP+ catalytic domain (aa. 390–420). Although this region is not a hinge, it was very susceptible to ligand binding due, in part, to its close connectivity with the G6PD::6PGL fusion region (aa. 450–500). Our data suggest that this region is susceptible to conformational changes in G6PD and 6PGL domains. These results agree with the previous report by Morales-Luna et al. [35], which has addressed this region’s role in the turnover and stability of fused enzyme G6PD::6PGL enzyme.

### 2.6. Exposure of Tryptophan Residues as a Result of Changes in Molecular Motions 

**Figure 10:**
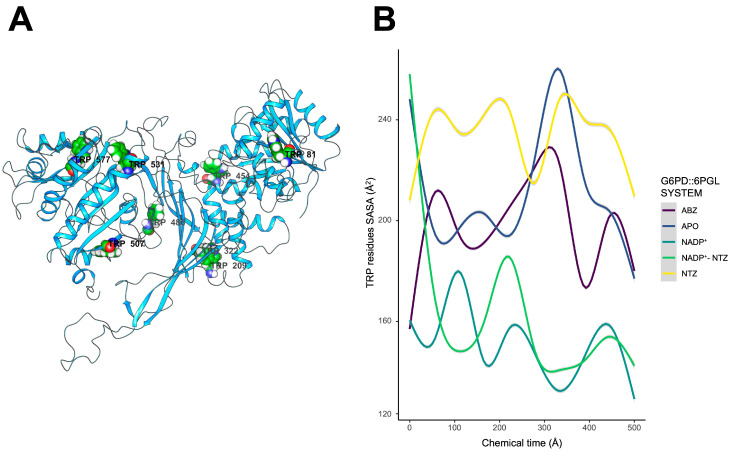
Surface-accessible solvent area (SASA) calculation of tryptophan residues. (**A**) Tryptophan residue localization on the GlG6PD::6PGL structure. (**B**) SASA time series calculation for all complexes during 500 ns of MDS.

Our MDS data show that interaction with antigiardial drugs may negatively impact the overall folding due, in part, to changes in molecular motions of GlG6PD::6PGL regions. Solvent exposure of indole moieties of tryptophan residues is very sensitive to surrounding microenvironmental conditions. Therefore, changes in solvent exposure of indole moieties of tryptophan residues can indicate changes in protein conformation, protein–protein interactions, and ligand binding [37]. Kelkar et al. showed that, for α-lactalbumin, tryptophan residues experience motionally restricted environments due, in part, to restraints on the reorientation of the water solvent around the excited-state tryptophan [38]. Therefore, the characterization of tryptophan dynamics can shed light on protein–ligand interactions, and aid in the development of new drugs [39]. The data showed that the experimentally determined changes in the protein structure cannot be completely tracked by MDS, suggesting that the interaction of NTZ can generate a substructure of G6PD::6GPL that is energetically restricted and dependent of more simulation time. However, to gain insight in the possible changes in the intrinsic fluorescence, the surface-accessible solvent area was calculated for all tryptophan residues (Figure 10). Moreover, endpoint free energy calculations were applied to evaluate the required work to expose the sidechain indole tryptophan group to the solvent.

**Figure 11:**
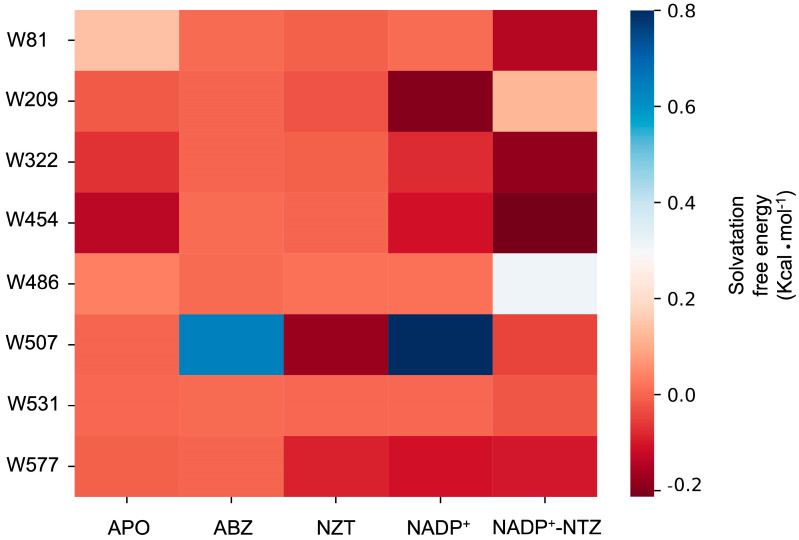
MMGBSA calculations of tryptophan solvation free energy of GlG6PD::6PGL. All computed energies are expressed as mean, and include the entropy calculated via normal mode analysis. Energies are presented in kcal·mol^−1^ at a temperature of 298 K.

The calculated SASA for NADP^+^ and NADP^+^–NTZ displayed the lowest values (~150–160 Å^2^). ABZ and the APO state of GlG6PD::6PGL showed higher SASA values, possibly due to the increasing motion of W81, W531, and W454 of the binding sites. In contrast, NTZ complexes displayed the highest SASA values for all tryptophan residues, with average values of ~220 Å^2^. NADP^+^ likely imposes restrictions on the motion of tryptophan residues, which can be coupled to the catalytical reorganization of the environment around the cofactors (Figure 11). The free energy of solvation can be used as a measure of stability, and can help us to understand the energetic requirements of structural changes. In these terms, endpoint free energy calculations upon the complete trajectories are useful for comparison of the feasibility of tryptophan solvation. In the presence of NADP^+^, ABZ, and APO complexes, more positive values were recorded for W81, W486, W507, and W531. For complexes containing NTZ only, the W507 free energy of solvation drastically decreased, suggesting that changes in protein motions can facilitate the solvation of this residue. Interestingly, for complexes in were NADP^+^ and NTZ are present in the simulation system, W81, W322, and W454 showed the most negative values for solvation, suggesting that those positions can encounter multiple stable conformations (Figure 11).

Interestingly, W507 is located 7 Å away from the NADP^+^ cofactor in an anticorrelated region that moves independently of the cofactor binding site. However, in the presence of NTZ, the independent motion of this region is lost, and regions in close contact with the cofactor site become strongly correlated. Even in the presence of NADP^+^, NTZ complexes display a propensity for an increase in the solvation free energy for this residue. Therefore, perturbations in the cofactor vicinity, such as those associated with interactions of NTZ, can promote relevant changes in the stability of protein, compromising protein activity and subsequent conformational stability (Figure 9).

### 2.7. Enzyme Activity of Cellular GlG6PD::6PGL 

**Figure 12:**
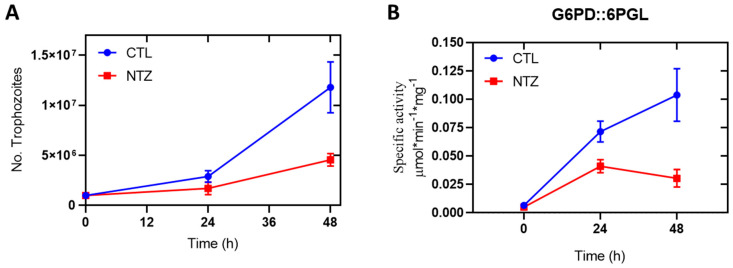
Cytotoxic effect of NTZ (**A**) and concomitant inhibition of cellular GlG6PD::6PGL (**B**). Data on the viability of *G. lamblia* and the activity of GlG6PD::6PGL. Cultures of *G. lamblia* trophozoites were exposed to a 4 µM concentration of NTZ. Values are presented as means ± SD from at least three independent experiments.

Several studies have demonstrated the in vitro antigiardiasic activity of NTZ [40]; the most widely accepted mechanism of NTZ is the disruption of energy metabolism by inhibiting the PFOR reaction cycle [22,27,41]. In the present study, we demonstrated that NTZ is a potent inhibitor of recombinant enzyme GlG6PD::6PGL, in addition to the previously described mechanism. Therefore, we were interested in determining whether NTZ also affects the activity of the G6PD::6PGL enzyme in a *G. lamblia* trophozoite culture. As shown in Figure 12, incubation of *G. lamblia* trophozoites with NTZ (4 µM) reduced the viability and specific activity of cellular GlG6PD::6PGL in a dose-dependent manner. Trophozoite counts were 62% lower after 48 h of culture in the presence of NTZ (Figure 12A) compared to culture in the absence of NTZ, whereas the specific activity of the GlG6PD::6PGL enzyme was decreased by around 71% in the presence of NTZ after 48 h (Figure 12B).

This effect may be partially explained by the importance of the GlG6PD::6PGL enzyme for cell metabolism, as it contributes to the nucleotide and lipid biosynthesis pathways, as well as the redox balance. As a microaerophilic pathogen, *G. lamblia* not only has to contend with toxic oxygen diffusion into the cell from the surrounding host tissue, but also with reactive oxygen species (ROS), such as H_2_O_2_ and nitric oxide (NO), and reactive nitrogen species, such as peroxynitrite (ONOO^-^), which originate in the host’s immune system [42]. Therefore, in order to prevail, the ROS must be eliminated—a mechanism enacted through water reduction [43,44]. NADPH-dependent oxygen reduction has been observed in *G. lamblia*, [12,45], for which NADPH molecules are necessary, as reducing power is transferred to the catalytic site of TrxR through its flavin adenine dinucleotide (FAD) cofactor. The production of NADPH in the pentose phosphate pathway is a primary antioxidant defense mechanism available to *G. lamblia* via the catalytic activity of G6PD::6PGL, and its inhibition would be lethal for parasites.

## 3. Materials and Methods

### 3.1. Chemicals

Nitazoxanide (NTZ), metronidazole (MTZ), and albendazole (ABZ) were acquired from Sigma Aldrich (St. Louis, MO, USA) to perform the inactivation assays. Nicotinamide adenine dinucleotide phosphate disodium salt (NADP^+^) (Roche, Basel, Switzerland) and D-glucose-6-phosphate disodium salt (G6P) (Sigma Aldrich, St. Louis, MO, USA) were used for activity assays.

### 3.2. Purification of the Recombinant Protein GlG6PD::6PGL 

Inactivation analysis was carried out with the recombinant GlG6PD::6PGL protein from *G. lamblia*, which was overexpressed in a bacterial heterologous system (*E. coli* strain BL21(DE3)Δzwf::kan^r^) containing the plasmid with the *glg6pd::6pgl* gene inserted. The cultures were induced with 300 nM of isopropyl-β-D-1-thiogalactopyranoside (IPTG) (Thermo Fisher Scientific, Hudson, NH, USA) and incubated for 18 h at 25 °C in nutrient-rich media called Luria Bertani (LB) culture medium. Subsequently, bacteria cells were harvested by centrifugation, suspended in lysis buffer (50 mM Tris, 150 mM NaCl, 0.5 mM phenylmethylsulfonyl fluoride (PMSF), and 1.4 mM β-mercaptoethanol; pH 8), and disrupted by sonication. The recombinant protein was purified in one step, by the immobilized nickel metal affinity chromatography method using ProfinityTM IMAC resin (Biorad, Hercules, CA, USA) and, subsequently, following the method employed in [12]. Protein activity of the G6PD domain was monitored spectrophotometrically by measuring the reduction in NADP^+^ at 340 nm utilizing a standard reaction mix (100 mM Tris–HCl buffer, 30 mM MgCl_2_, 250 µM glucose-6-phosphate, and 250 µM NADP^+^; pH 8.0). Purified GlG6PD::6PGL protein was resolved in 12% SDS–PAGE gel, and visualized by staining with Coomassie Brilliant Blue (R-250) (Sigma-Aldrich, St. Louis, MO, USA). Purified protein concentration content was quantified by the Lowry method [46], using bovine serum albumin (BSA) as the standard.

### 3.3. Inactivation Assays of Fused G6PD::6PGL by NTZ 

#### 3.3.1. In Vitro Analysis of Fused GlG6PD::6PGL Inactivation with Antigiardial Drugs 

The inhibitory concentration 50 of NTZ was determined, as previously reported [23,47,48], to reduce the activity of the GlG6PD::6PGL, and compared to the activity of MTZ (a nitro antiparasitic drug, the mechanism of action of which is due to PFOR). The drug ABZ (an inhibitor of microtubule polymerization by selective binding to the β-tubulin subunit) was also included in this assay. To determine the activity, the fused G6PD::6PGL was adjusted to 0.2 mg/mL and incubated with NTZ, MTZ, and ABZ compounds at increasing concentrations (0–500 µM) for 2 h at 37 °C. Subsequently, the activity of the G6PD domain was determined as mentioned above, and the results were expressed as the percentage of residual activity against the concentration of each compound.

#### 3.3.2. Second-Order Rate Constant (*k*_2_) of NTZ

The binding of NTZ to G6PD::6PGL (enzyme-inhibitor complex) was evaluated according to the second-order rate constant (*k*_2_). To this end, the pseudo-first-order inactivation constants (*k*_1_) were determined, where the protein concentration of the GlG6PD::6PGL was adjusted to 0.2 mg/mL and then incubated at 37 °C with different concentrations of compound (0–500 µM). The residual activity was monitored in time intervals (0–120 min), and expressed as a percentage of activity against the incubation time. The results were fitted to a mono-exponential decay model using the following equation: $A_{R}=A_{0}\;e^{-kt}$, where A_R_ is the residual activity at different times, A_0_ is the activity at the initial time, and *k* is the pseudo-first-order inactivation constant (min^−1^) [48,49]. Subsequently, the second-order rate constant (*k*_2_) was obtained by fitting the *k*_1_ constants against the compound concentration by linear regression.

### 3.4. Structural Analysis by Intrinsic and Extrinsic Fluorescence Assays 

To determine the effect of the compounds on the tertiary structure of the GlG6PD::6PGL protein, intrinsic fluorescence and extrinsic fluorescence assays were performed with 8-anilinonaphthalene-1-sulfonic acid (ANS). Both assays were performed in a Perkin-Elmer LS-55 fluorescence spectrometer (Perkin Elmer, Wellesley, MA, USA) equipped with a quartz cell and a path length of 1 cm [23,48,49,50]. In two assays, the protein was adjusted at 0.1 mg/mL and incubated for 2 h at 37 °C with a concentration of 100 µM of each antigiardial drug (NTZ, MTZ, and ABZ) in a buffer containing 50 mM K_2_HPO_4_, pH 7.35. Fluorescence decay data were obtained using an excitation wavelength of 295 nm for the intrinsic fluorescence, with excitation and emission slits of 4.0 nm and 5.0 nm, respectively. The fluorescence spectrum was acquired in the range of 300 to 500 nm. For the extrinsic fluorescence assay (ANS), the ANS reagent was exited at 395 nm, and the emission was collected in the range of 400 to 600 nm, with excitation and emission slits of 5.0 and 5.0 nm, respectively. For both fluorescence assays, the spectra of the blanks were subtracted from the experimental samples containing the respective protein.

### 3.5. Molecular Docking Study 

Molecular docking assays were performed to characterize the binding of NTZ (inhibitor) versus ABZ (not inhibitor) on GlG6PD::6PGL. The molecular docking was performed without the presence of NADP^+^, and the compound structures were downloaded from the PubChem compounds database and submitted to geometric optimization employing the MMFF94x force field in Avogadro [51]. Docking calculations were performed using the Swiss dock server [52]. The best binding mode of each molecule was selected based on the lowest binding free energy and the largest cluster size.

### 3.6. Molecular Dynamics Simulations of GlG6PD::6PGL 

Molecular dynamics simulations (MDS) were carried out to examine the intermolecular association between GlG6PD::6PGL and ABZ or NTZ drugs. The starting atomic coordinates were retrieved from docking assays. The OPLS-AA force field from the Schrödinger Maestro 2020-4 suite (New York, NY, USA) was applied to explicitly pronate the system at pH 7, with the assistance of *propKa* [53]. Protein and drug parameters derived from the ff14SB [54] and GAFF [55] force fields for protein and organics molecules. GlG6PD::6PGL complexes were solvated in a orthorhombic space with a length of 15 Å, using as solvent the explicit model of water TIP3P. The simulation systems comprised ~143,538 atoms, with ~43,925 water molecules, 0.15 M NaCl, and 742 protein residues. All MDS were performed using the pmemd.cuda [56,57] module within the Amber20 package (https://ambermd.org/, accessed on 13 January 2023) [58]. The initial minimization process comprised 30,000 steps in combination of the steepest descent and the conjugate gradient methods. The heating protocol was executed in six stages under the NvT ensemble, with the following temperature and duration settings: 10 K for 500 ps, 100 K for 1000 ps, 200 K for 1000 ps, 300 K for 1000 ps, and 400 K for 500 ps. Subsequently, the system was cooled to 300 K for 2500 ps, utilizing linear interpolation between adjacent time points. A mixed equilibration scheme was applied under the NpT ensemble at 300 K. Pre-equilibration consisted of 50,000 ps with solute molecules restrained by a 5 Kcal mol^−1^ force constant, followed by 50,000 ps of equilibration under unrestrained conditions. Additionally, 100 ns of conventional molecular dynamics (cMD) were simulated under the NpT ensemble at a constant pressure of 1 atm, employing a Monte Carlo barostat. The particle mesh Ewald (PME) method was used to compute long-range electrostatic interactions (tolerance of 1 × 10^−9^) in the periodic systems at an integration time of 2 fs, with the SHAKE algorithm enabled to constrain all bonds involving hydrogen during simulations. Weak coupling was used to apply the temperature to an external bath temperature coupling at 303 K.

Gaussian accelerated molecular dynamics is an enhanced sampling technique designed to smooth biomolecular potential energy surfaces and reduce energetic barriers between processes in biomolecular systems [36]. This method adds a harmonic boost potential to dihedral angles, as well as directly to potential energy, promoting the appearance of a time-dependent molecular process that requires a considerable chemical simulation duration [59,60,61]. The initial GaMD preparation stage included an additional 50 ns to collect variables and production stages of 500 ns, with sigma0P = 6.0 and sigma0D = 6.0 and the same simulated conditions as conventional MDS.

### 3.7. Trajectory Analysis

All structural analyses were carried out with CPPTRAJ v. 5.0 [62] and the VMD package [63]. Root mean square deviation (RMSD), root mean square fluctuation (RMSF), and radius of gyration (Rog) were determined to evaluate the dynamic behavior of the complexes during the simulation run; RMSD and Rog metrics were calculated for alpha carbon, and RMSF was calculated for beta carbon atoms during all simulation steps.

The dynamic cross-correlation coefficient maps (DCCMs) were calculated for all complexes, sampling residue pairs correlations as a time-averaged vector. The DCCMs were generated using Bio3D [64] along all simulation trajectories for each complex.

As a measurement of solvent exposure, the solvent-accessible surface area (SASA) of tryptophan residues was calculated with the LCPO method at time steps. The MMGBSA method was employed to calculate the solvation free energy associated with the exposure of each tryptophan residue. MM/GBSA binding free energy calculations were carried out to estimate the strength of tryptophan solvation in each of the complexes. Free energies were calculated using all snapshots retrieved from the 500 ns of each MD production, using the MMPBSA.py [65] module in Amber20. GB calculations were carried out using the modified GB model (igb = 2) with mbondi2 and default α, β, and γ values. Dielectric constants for the solvent and the solute were set to 80 and 5, respectively. As reported earlier, a salt concentration of 0.15 M was considered a mimic of physiological conditions. Entropic contributions to binding free energy estimates were included for all systems derived from normal mode calculations over 15,000 time steps.

### 3.8. Enzyme Activity of Cellular GlG6PD::6PGL 

*Giardia lamblia* WB strain (ATCC number 30957) was grown in Keister’s modified TYI-S-33 medium, supplemented with 10% fetal bovine serum (FBS). Confluent cells monolayer were placed on ice for 20 min, then collected by centrifugation at 3500× *g*. Eppendorf tubes with 1.5 mL of medium and 4 mM of nitazoxanide were inoculated with 1.5 × 10^5^ trophozoites of *G. lamblia,* and subsequently incubated at 37 °C. A control medium with trophozoites was also included. After incubation, the trophozoites were centrifuged at 2755× *g* for 5 min at 4 °C; the supernatant was discarded, and the pellet was resuspended in phosphate-buffered saline (PBS), and this procedure was repeated two times. Next, the trophozoites were resuspended in a lysis buffer (50 mM KH_2_PO_4_, 150 mM NaCl, 2 mM DTT, 0.5 mM phenylmethylsulfonyl fluoride (PMSF), and 1.4 mM β-mercaptoethanol; pH 7.35). Then, the cells were sonicated with 15 pulses of 45 s, with rest intervals of 2 min. Next, the lysate was centrifuged at 14,500× *g* for 30 min, and the supernatant was collected. Enzyme activity was spectrophotometrically measured at 25 °C following absorbance at 340 nm during the first 5 min of recording; the data are reported as nmol of NADP^+^ reduced min^−1^ mg^−1^ protein^−1^.

## 4. Conclusions

Polypharmacology refers to the interaction of drugs with different targets, which may affect important pathways related to disease or organismal metabolism. Therefore, polypharmacological studies may identify new off-targets for existing drugs. In this work, we evaluated the effect of nitazoxanide on the G6PD::6PGL protein of *G. lamblia*. Our results of inhibition, molecular docking, and molecular dynamics simulations indicate that NTZ could conceivably be a molecular target for GlG6PD::6PGL enzymes. Our results also suggest explanations of other mechanisms by which NTZ may exert therapeutic effects through an imbalance in redox maintenance and inhibition of microorganismal proliferation. Therefore, we propose G6PD::6PGL as a new pharmacological target for the antiparasitic drug nitazoxanide.

## Data Availability

Not applicable.
